# Psoriasis and Cardiovascular Risk—Do Promising New Biomarkers Have Clinical Impact?

**DOI:** 10.1155/2017/7279818

**Published:** 2017-08-29

**Authors:** Sirje Kaur, Külli Kingo, Mihkel Zilmer

**Affiliations:** ^1^Clinic of Dermatology, Institute of Clinical Medicine, University of Tartu, Tartu, Estonia; ^2^Clinic of Dermatology, Tartu University Hospital, 31 Raja St., 50417 Tartu, Estonia; ^3^Institute of Biomedicine and Translational Medicine, Department of Biochemistry, The Centre of Excellence for Genomics and Translational Medicine, University of Tartu, 19 Ravila St., 50411 Tartu, Estonia

## Abstract

Epidemiological studies suggest an increased prevalence of cardiovascular disease (CVD) in patients with psoriasis (PS). Therefore, emphasis has lately been laid on the necessity for clinical evaluation of the risk of CVD in these patients. The systemic inflammatory markers C-reactive protein (CRP) and interleukin- (IL-) 6, which have long been used to predict future CVD in the general population, are increased manyfold in patients with PS. Lipid abnormalities characterized by elevated triglycerides, low HDL cholesterol, and higher concentrations of LDL cholesterol and its oxidized form are also prevalent in patients. There is a need for additional laboratory markers for the assessment of cardiovascular status of patients with PS. Due to frequent comorbid overweight and obesity, biologically active compounds produced by adipocytes may have an impact on monitoring the status of the cardiovascular system of patients with PS. For this purpose, two adipokines, adiponectin and leptin, have been most extensively studied. The review focuses on some inflammatory and oxidative stress aspects in patients with PS through the analysis of the impact of prominent adipokines and oxidized low-density lipoprotein (oxLDL) to assess their eligibility for clinical practice as markers of CVD risk in patients with PS.

## 1. Introduction

Several lines of evidence indicate that psoriasis (PS) is associated with enhanced atherosclerosis and risk of cardiovascular disease (CVD) [1–3] such as coronary artery disease, ischemic heart disease, and myocardial infarction [4]. The results of epidemiological studies have also demonstrated that the risk to develop CVD is higher in patients with severe PS [5–9] and that the risk persists after adjusting for conventional cardiovascular risk factors such as obesity, hypertension, diabetes, and smoking [1, 8].

Central obesity, a component of metabolic syndrome (MS), is frequently encountered in patients with PS, often in association with other components of this syndrome such as insulin resistance, hypertension, and dyslipidemia [10–12]. Large epidemiological studies have demonstrated that patients with moderate to severe PS have about twice as high prevalence and incidence of obesity as compared to the general population [10, 13, 14]. According to several authors, the severity of obesity positively correlates with the Psoriasis Area and Severity Index (PASI) [15–19]. The discussion, whether obesity is a result or a cause of PS and future cardiovascular events, is still open [7, 20].

The pathogenesis of atherosclerosis followed by CVD is inflammation dependent [1, 21, 22]. Therefore, an increased prevalence of atherosclerosis in patients with PS is mostly explained by a chronic systemic inflammation [1]. However, it is still unclear whether PS itself or some other risk factors, for example genetic and lifestyle factors, are responsible for the increased prevalence of atherosclerosis in patients with PS [8, 9].

The explanation of associations between PS and CVD is further complicated by the fact that traditional systemic antipsoriatic treatments such as acitretin, cyclosporine, and corticosteroids may contribute to the development of CVD risk factors such as hypertension, obesity, diabetes, and dyslipidemia [8] whilst anti-TNF-*α* treatment does not have this side effect [23].

Epidemiological studies have shown that due to comorbidities, especially CVD, the life expectancy of patients with severe PS is 3 to 5 years shorter as compared to that of nonpsoriatic controls [24–26]. Therefore, several recent studies have emphasized the necessity for additional laboratory markers for earlier diagnosis of cardiovascular complications and their follow-up [14]. Ideally, these markers should also correlate with the severity of PS estimated by PASI or body surface area (BSA). This short review focuses on some inflammatory and oxidative stress aspects of PS, considering also the potential of prominent adipokines and oxidized low-density lipoprotein (oxLDL) as markers of cardiovascular status and skin disease severity in patients with PS.

## 2. Search Strategy

We searched medical databases such as PubMed, Wiley Online Library, and Web of Science for English language articles published between 2003 and 2017 by entering the terms “psoriasis,” “cardiovascular disease,” “obesity,” “body mass index (BMI),” “interleukins,” “adipokines,” “C-reactive protein,” and “oxidized LDL.” The inclusion criteria for this review comprised clinical studies and reviews, which focused on the associations between the search terms. The reference lists of articles were searched for additional publications. Potentially relevant studies were printed out and read in full text to be included in this review.

## 3. Inflammatory Background of Psoriasis and Obesity

The underlying mechanisms that link PS and atherosclerosis with the subsequent CVD are not well understood; however, several lines of evidence suggest that a chronic inflammation with the involvement of humoral and cellular immunity may contribute to both diseases [27, 28]. Nowadays, PS is interpreted as the outcome of an inappropriate immunocyte (mainly T cell)-based activation event to an unknown antigen [29]. During inflammation, the communication between epidermal keratinocytes, dermal vascular cells, and immunocytes, including activated antigen presenting cells and T memory/effector cells, is driven by cytokines, chemokines, adhesion molecules, and receptors [29–31]. Pathogenetic T cells are mainly represented by Th17 and Th1 lymphocytes [2, 32–34]. Accordingly, elevated serum levels of Th1-derived cytokines such as interferon- (IFN-) *γ*, tumor necrosis factor- (TNF-) *α*, and interleukin- (IL-) 2 have been detected in the sera of patients [32, 35–37]. IFN-*γ* and TNF-*α* not only induce keratinocytes to produce several other cytokines, for example IL-6 and IL-8 [2], but also increase the expression of intercellular adhesion molecule- (ICAM-) 1 that promotes the infiltration of T cells and other inflammatory cells [32].

Similarly to PS, subclinical inflammation induced by obesity is characterized by increased production of inflammatory cytokines IL-6 and TNF-*α* and higher C-reactive protein (CRP) levels [38–40]. There are also some other coinciding pathological pathways between PS and obesity, for example, both disorders are associated with systemic high-grade oxidative stress (OxS) and the related pathophysiological outcomes, including the promotion of LDL oxidation [11, 41, 42]. PS combined with obesity/MS is likely to produce a higher degree of systemic inflammation and OxS [43], promoting endothelial dysfunction and the formation of atherosclerotic plaques. These pathological events may activate or intensify preexisting atherosclerosis and result in the development of significant CVD [8, 44].

## 4. CRP and Cytokines

Previous studies have demonstrated that PS and obesity share common inflammatory mediators such as CRP and IL-6. Both IL-6 and CRP, the latter measured by highly sensitive assay (hsCRP), have been found to be associated with subclinical atherosclerosis [4, 45] and, therefore, have a predictive value for future cardiovascular events [45]. Both these markers are overexpressed in psoriasis [27, 46–49], including in patients without overweight/obesity or other traditional CVD risk factors such as hypercholesterolemia, hypertension, and diabetes [26, 50]. The findings of the studies included in this review comparing the levels of inflammatory markers and adipokines in patients with PS and nonpsoriatic controls are summarized in Table 1. The association between the levels of CRP and IL-6 with the severity of psoriatic inflammation is confirmed by the significant decrease of IL-6 levels during both light and systemic antipsoriatic therapy with corticosteroids and methotrexate [39, 51, 52]. During infliximab monotherapy, inflammatory markers decreased in concordance with the decrease of PASI [53]. Therefore, several authors are in agreement that PS may be an independent CVD risk factor [3, 47]. Thus, the levels of these inflammatory markers should be measured regularly in patients with PS as well as other chronic inflammatory diseases to discover an enhanced risk of developing CVD. An increase in hsCRP also reflects metabolic disorders, including insulin resistance and adiposity [45], which are common in patients with PS. Using hsCRP and IL-6 for the assessment of PS activity is questionable. To date, no single biomarker has been found to correlate with PASI consistently. For example, the positive correlations of PASI with CRP and IL-6 were demonstrated by Coimbra et al. [52, 54, 55], whilst others found no correlation between PASI and the levels of CRP or IL-6 [49, 56] (Table 1). In our previous work, we found a linear correlation between PASI and IL-6 [57], but not in the group of patients investigated a year before [50].

The suppression of systemic inflammation by means of antipsoriatic therapy could decrease the risk of CVD that should be accompanied by the decrease in CRP and IL-6 levels. According to Coimbra et al. [55], after 12-week phototherapy with narrow-band UVB (NBUVB) and psoralen + UVA (PUVA), CRP in patients decreased significantly (*P* ≤ 0.01) compared to that of the pretreatment value while topical therapy did not influence significantly the blood levels of inflammatory markers [58]. The authors also reported a decrease in the levels of several inflammatory cytokines involved in the pathogenesis of PS (IL-22, IL-17, IL-23, IL-8, TNF-*α*, and vascular endothelial growth factor) after 12-week phototherapy [59]. The decrease of CRP after treatment is an evidence of decreased systemic low-grade inflammation implicated in atherosclerosis.

## 5. Adiponectin and Leptin

After the discovery that adipose tissue is not a simple storage of energy but metabolically an extremely active tissue, adipocyte-derived bioactive compounds termed adipokines gained enormous attention. Through the release of adipokines, adipose tissue regulates food intake, energy expenditure, insulin sensitivity, and inflammation [38, 60]. The pattern of secretion of adipokines mirrors adipose tissue function, and this pattern is important to establish the individual risk of developing metabolic and cardiovascular comorbidities of obesity [61]. Mainly two adipose tissue mass-dependent adipokines—adiponectin (ADIPO) and leptin—have attracted the attention of scientists as possible mediators of CVD risk associated with obesity [62, 63].

There are several reasons why ADIPO and leptin may serve as diagnostic or prognostic biomarkers of CVD. ADIPO has insulin-sensitizing, anti-inflammatory, and atheroprotective effects [37, 62]. Decreased circulating levels of ADIPO have been associated with higher levels of inflammatory cytokines and increased OxS in well-known risk factor of CVD such as obesity [64]. Reduced levels of ADIPO are associated with insulin resistance and several vascular adverse events, including impaired endothelium-dependent vasodilatation, impaired ischaemia-induced neovascularization, and diastolic heart failure [65–68]. ADIPO levels inversely correlate with serum CRP in obese subjects, in diabetics and in those affected by coronary artery disease [69].

Leptin, having structural and functional similarities with proinflammatory cytokines IL-6 and IL-12 [38], exerts proinflammatory and proatherogenic effect through mechanisms involving endothelial cell activation and thrombogenesis [70]. The effects of leptin also include the accumulation of reactive oxygen species (ROS) in endothelial cells and stimulation of vascular smooth muscle cell proliferation, acceleration of vascular calcification, and enhancement of platelet adhesiveness [71]. CVD risk factors (central obesity, insulin resistance, and dyslipidemia) are associated with decreased ADIPO and increased leptin levels [37, 44].

While there are conflicting reports, most of the recent studies have demonstrated significantly decreased ADIPO or its high molecular form levels in patients with PS as compared to those of the healthy controls [37, 43, 72], especially in patients with moderate and severe disease [54] (Table 1). According to Baran et al. [43], ADIPO levels in patients with PS rose with the increase in disease activity, expressed by PASI score. In patients with normal body weight, an increase in ADIPO concentration has been reported [44, 50, 73]. It might be possible that the increase in ADIPO levels in high-risk population with advanced atherosclerosis or other chronic inflammatory conditions is part of a compensatory mechanism to limit further endothelial damage [74, 75]. Consequently, in patients with chronic inflammatory diseases, the upregulation of expression, synthesis, and release of ADIPO may occur due to increased systemic inflammation, accompanied by higher levels of TNF-*α* and other cytokines. ADIPO has been shown to have a counter-regulatory action on TNF-*α* production [38]. Obesity may counteract this compensatory mechanism because the raised levels of TNF-*α* and IL-6 in obesity suppress ADIPO production by adipose tissue [76, 77]. Thus, ADIPO mediates protective effects in obesity-related metabolic and vascular diseases, presumably by its anti-inflammatory action. As ADIPO levels are mainly associated with body weight and decrease in obesity, this attractive marker might be used for the assessment of general CVD risk in obese and overweight individuals to follow their progression to impaired glycose tolerance and CVD.

Leptin has an important role in the central regulation of food intake and energy expenditure, with low leptin levels driving increased food intake and reduced energy expenditure and increased leptin levels promoting decreased food intake and increased energy expenditure [78]. Leptin concentrations increase with the increase in body mass and inflammatory activity [77]. Conditions associated with the release of proinflammatory IL-6 and TNF-*α* are known to increase leptin synthesis [71]. In the cardiovascular system, leptin actions are potentially proatherogenic, prothrombotic, and angiogenic [79–81]. Interestingly, in obese individuals who have elevated leptin levels, only the anorectic effect of leptin is impaired, whereas other effects are maintained, a phenomenon known as selective leptin resistance [78, 81]. Thus, hyperleptinemia contributes to atherogenesis in these patients. Hyperleptinemia is associated with the impairment of NO-dependent vasorelaxation, an increase in OxS as well as in the level of endothelin (a potent vasoconstrictor) [81]. All these features are markers of endothelial dysfunction, the early stage in atherogenesis. Leptin and the CVD risk marker CRP have been found to be independently associated [71].

In several studies, plasma leptin levels in patients with PS have been observed to be higher than those of the healthy controls [13, 82, 83]. However, some other studies have reported that plasma leptin levels in patients were not different from the controls [37, 73]. There are several potential explanations for these differing results. Most of the studies had a relatively small number of patients with highly different PASI and body mass index (BMI) values. In addition, different measurement methods were used. According to Coimbra et al. [72], the increase in leptin levels in patients with PS is usually associated with overweight and obesity, not with PS severity. Considering that leptin has been shown to promote important links in the pathogenesis of PS such as cytokine secretion, keratinocytes proliferation, and angiogenesis [72], more studies are needed to characterize the relationship between leptin (or ADIPO) levels and PS. However, due to their multiple roles in inflammation, insulin resistance, diabetes, atherosclerosis and obesity, and possibility of other forms of adipokine-resistance in addition to selective leptin resistance in appetite regulation, adipokines have not yet been approved as prognostic markers of CVD in the general population [84, 85].

Whether adipokines could be used as biomarkers of comorbid CVD in patients with PS is not known. There are currently limited data concerning the changes in adipokine levels after treatment. ADIPO levels increased after 12-week phototherapy in comparison to their levels before treatment (*P* ≤ 0.01) which was demonstrated by Coimbra et al. [55, 58].

## 6. Dyslipidemia and OxLDL

The principal CVD risk factors are hypertension and dyslipidemia; the latter is characterized by elevated levels of total cholesterol, low-density lipoprotein (LDL) and triglycerides, and lower levels of high-density lipoproteins (HDL) [86]. These changes are found consistently in patients with PS [47, 54, 58, 87]. Inflammatory mediators, including adipocyte-derived cytokines such as TNF-*α*, IL-6, and leptin, are known to induce dyslipidemia [7]. Therefore, proinflammatory activities observed in PS may initiate abnormalities in plasma lipid/lipoprotein levels [88]. As PS is frequently associated with obesity, the excess adipose tissue might further contribute to atherogenic dyslipidemia [7]. In fact, several studies have shown the common features of atherogenic dyslipidemia with increased blood levels of total cholesterol, triglycerides, LDL, and apolipoprotein A and low HDL and apolipoprotein B levels in patients with PS [7, 89].

In conditions of OxS that accompanies a chronic inflammatory disease, including PS, excess LDL is oxidatively modified. Oxidative modification of LDL is one of the earliest events in the pathogenesis of atherosclerosis [90], circulating levels of oxLDL and reflecting the intensity of oxLDL formation in the vascular wall [91] that can be followed by the rupture of atherosclerotic plaque and subsequent thrombosis [54]. OxLDL is a proinflammatory chemoattractant agent for macrophages and T lymphocytes and cytotoxic for endothelial cells and stimulates the release of soluble inflammatory molecules [92]. Thus, OxLDL, which contains hundreds of different oxidized lipid molecules, can be considered as a hallmark of hyperlipidemia and atherosclerosis [92].

OxLDL levels are not frequently estimated in patients with PS. A few studies have demonstrated increased levels of oxLDL in patients with a significant association with BMI [50, 54]. Significant association between circulating oxLDL and BMI was also found [54]. OxLDL binds to *β*_2_-glycoproteins (*β*_2_-GPI) to form oxLDL-*β*_2_-GPI complexes in the intima, and these complexes are released into circulation [90]. Although the roles of oxLDL-*β*_2_-GPI and their antibodies are still controversial, it has been postulated that *β*_2_-GPI binds oxLDL to neutralize its proinflammatory and proatherogenic effect [93]. The occurrence of oxLDL-*β*_2_-GPI has been connected to the chronic inflammation of the vasculature and OxS, and these complexes have been found in patients with systemic autoimmune disorders such as systemic lupus erythematosus, systemic sclerosis, and type 2 diabetes mellitus [90, 94, 95], but also in patients with PS [50]. In addition, the skin of patients with PS has shown positive oxLDL staining compared to sex- and age-matched healthy volunteers, whereas there was no staining in nonlesional skin samples from the patients [96]. The study of Coimbra et al. has demonstrated that topical treatment with calcipotriol or betamethasone dipropionate, or a combination of the two had no effect on oxLDL plasma level [58]. OxLDL levels in patients, who were treated with 12-week phototherapy, have improved but remained higher as compared to healthy controls [58]. In patients with PS, regular lipid screening is important, especially when treated with drugs causing hyperlipidemia such as acitretin and cyclosporine [23]. Therefore, the assay for the estimation of the level of oxLDL in patients with PS would be of value, especially in obese patients, but it has not yet been approved as a biochemical prognostic marker for atherosclerotic disease.

## 7. Conclusions

So far, no laboratory markers have been employed to evaluate psoriasis activity, although such a marker would be highly valuable to monitor the effect of therapy and predict recurrences. Psoriasis and obesity are both characterized by overexpression of key proinflammatory cytokines, hsCRP and IL-6, which have been accepted as classical markers of CVD risk and are therefore valuable markers for the assessment of the cardiovascular status of patients with PS. Although the amount of scientific information concerning the levels of adipokines is steadily increasing, further studies are warranted to address the role of adipokines as clinical biomarkers. Additional research is required to establish the perspective for oxLDL as a biomarker of increased cardiovascular risk.

## Figures and Tables

**Table 1:** Selected hematological parameters in patients with psoriasis as compared to nonpsoriatic controls and their relation to the Psoriasis Area and Severity Index (PASI).

Author, year [ref.]	Number of patients/controls	Mean/median PASI or BSA	*P*	Correlation to PASI
C-reactive protein				
Chodorowska et al., 2004 [51]	175/30	29.2	*P* < 0.001**↑**	ND
Coimbra et al., 2010 [52]	73/38	18.0	*P* < 0.001** ↑**	In correlation with PASI
Takahashi et al., 2014 [49]	97/79	10.6/9.2 (male/female)	*P* < 0.05** ↑**	Not associated with PASI
Vachatova et al., 2016 [44]	74/65	Median 15.3	*P* < 0.001** ↑**	ND
Interleukin-6				
Coimbra et al., 2010 [55]	66/37	18.8	*P* < 0.01** ↑**	In correlation with PASI
Deeva et al., 2010 [56]	35/10	8.7	*P* < 0.0001**↑**	Not associated with PASI
Kaur et al., 2012 [57]	58/58	9.5	*P* < 0.001** ↑**	In correlation with PASI
Adiponectin				
Coimbra et al., 2009 [54]	56/37	19.2	*P* = 0.001**↓**	ND
Gerdes et al., 2011 [73]	79/80	12.2	*P* = 0.0094 **↑**	Not associated with PASI
Kaur et al., 2011 [50]	60/44	10.1	NS (all patients)	ND
Li et al., 2014 [37]	122/134	Median BSA 2.9%^∗^	*P* < 0.001**↓**	ND
Baran et al., 2015 [43]	49/16	16.5	*P* = 0.004**↓**	Not associated with PASI
Vachatova et al., 2016 [44]	74/65	Median 15.3	NS	ND
LEPTIN				
Gerdes et al., 2011 [73]	79/80	Median 12.2	NS	Not associated with PASI
Kaur et al., 2011 [50]	60/48	10.1	NS (all patients)	Not associated with PASI
Li et al., 2014 [37]	122/134	Median BSA 2.9%^∗^	NS	ND
Baran et al., 2015 [43]	49/16	16.5	*P* = 0.002**↓**	Not associated with PASI
Vachatova et al., 2016 [44]	74/65	Median 15.3	*P* < 0.01** ↑**	ND
Coimbra et al., 2010 [55]	66/37	18.8	*P* < 0.001** ↑**	ND

PASI: Psoriasis Area and Severity Index. (the interpretation of PASI score differed between the studies. In most studies, the score below 10 was interpreted as mild disease, between 10 and 20 as moderate, and above 20 as severe disease.) BSA: body surface area; **↑**: increased in patients; **↓**: decreased in patients; NS: not significant; ND: not done; ^∗^mild disease.

## References

[1] Mehta N. N., Gelfand J. M. (2014). High density lipoprotein cholesterol function improves after successful treatment of psoriasis: a step forward in the right direction. *Journal of the American Academy of Dermatology*.

[2] Ghazizadeh R., Shimizu H., Tosa M., Ghazizadeh M. (2010). Pathogenic mechanisms shared between psoriasis and cardiovascular disease. *International Journal of Medical Sciences*.

[3] Boehncke W.-H., Boehncke S. (2012). Cardiovascular mortality in psoriasis and psoriatic arthritis: epidemiology, pathomechanisms, therapeutic implications, and perspectives. *Current Rheumatology Reports*.

[4] Bonaterra G. A., Zügel S., Kinscherf R. (2010). Novel systemic cardiovascular disease biomarkers. *Current Molecular Medicine*.

[5] Mallbris L., Akre O., Granath F. (2004). Increased cardiovascular mortality in psoriasis inpatients but not in outpatients. *European Journal of Epidemiology*.

[6] Gelfand J. M., Neimann A. L., Shin D. B., Wang X., Margolis D. J., Troxel A. B. (2006). Risk of myocardial infarction in patients with psoriasis. *The Journal of the American Medical Association*.

[7] Takahashi H., Iizuka H. (2012). Psoriasis and metabolic syndrome. *The Journal of Dermatology*.

[8] Pietrzak A., Bartosińska J., Chodorowska G., Szepietowski J. C., Paluszkiewicz P., Schwartz R. A. (2013). Cardiovascular aspects of psoriasis: an updated review. *International Journal of Dermatology*.

[9] Yeung H., Takeshita J., Mehta N. N. (2013). Psoriasis severity and the prevalence of major medical comorbidity: a population-based study. *JAMA Dermatology*.

[10] Neimann A. L., Shin D. B., Wang X., Margolis D. J., Troxel A. B., Gelfand J. M. (2006). Prevalence of cardiovascular risk factors in patients with psoriasis. *Journal of the American Academy of Dermatology*.

[11] Wakkee M., Thio H. B., Prens E. P., Sijbrands E. J. G., Neumann H. A. M. (2007). Unfavorable cardiovascular risk profiles in untreated and treated psoriasis patients. *Atherosclerosis*.

[12] Sterry W., Strober B. E., Menter A. (2007). Obesity in psoriasis: the metabolic, clinical and therapeutic implications. Report of an interdisciplinary conference and review. *The British Journal of Dermatology*.

[13] Versini M., Jeandel P. Y., Rosenthal E., Shoenfeld Y. (2014). Obesity in autoimmune diseases: not a passive bystander. *Autoimmunity Reviews*.

[14] Gisondi P., Giglio M. D., Girolomoni G. (2016). Considerations for systemic treatment of psoriasis in obese patients. *American Journal of Clinical Dermatology*.

[15] Wolk K., Mallbris L., Larsson P., Rosenblad A., Vingård E., Ståhle M. (2009). Excessive body weight and smoking associates with a high risk of onset of plaque psoriasis. *Acta Dermato-Venereologica*.

[16] Armstrong A. W., Harskamp C. T., Armstrong E. J. (2012). The association between psoriasis and obesity: a systematic review and meta-analysis of observational studies. *Nutrition & Diabetes*.

[17] Langan S. M., Seminara N. M., Shin D. B. (2012). Prevalence of metabolic syndrome in patients with psoriasis: a population-based study in the United Kingdom. *The Journal of Investigative Dermatology*.

[18] Ni C., Chiu M. W. (2014). Psoriasis and comorbidities: links and risks. *Clinical, Cosmetic and Investigational Dermatology*.

[19] Uczniak S., Gerlicz Z. A., Kozłowska M., Kaszuba A. (2016). Presence of selected metabolic syndrome components in patients with psoriasis vulgaris. *Advances in Dermatology and Allergology*.

[20] Boehncke S., Boehncke W. H. (2014). ‘Upgrading’ psoriasis responsibly. *Experimental Dermatology*.

[21] Steyers C. M., Miller F. J. (2014). Endothelial dysfunction in chronic inflammatory diseases. *International Journal of Molecular Sciences*.

[22] Roifman I., Beck P. L., Eisenberg M. J., Genest J. (2011). Chronic inflammatory diseases and cardiovascular risk: a systematic review. *The Canadian Journal of Cardiology*.

[23] Takeshita J., Grewal S., Langan S. M. (2017). Psoriasis and comorbid diseases: implications for treatment. *Journal of the American Academy of Dermatology*.

[24] Abuabara K., Azfar R. S., Shin D. B., Neimann A. L., Troxel A. B., Gelfand J. M. (2010). Cause-specific mortality in patients with severe psoriasis: a population-based cohort study in the U.K. *The British Journal of Dermatology*.

[25] Mehta N. N., Yu Y., Pinnelas R. (2011). Attributable risk estimate of severe psoriasis on major cardiovascular events. *The American Journal of Medicine*.

[26] Reich K. (2012). The concept of psoriasis as a systemic inflammation: implications of disease management. *Journal of the European Academy of Dermatology and Venereology*.

[27] Lubrano E., Cantini F., Costanzo A. (2015). Measuring psoriatic disease in clinical practice. An expert opinion position paper. *Autoimmunity Reviews*.

[28] Gerdes S., Osadtschy S., Buhles N., Baurecht H., Mrowietz U. (2014). Cardiovascular biomarkers in patients with psoriasis. *Experimental Dermatology*.

[29] Tonel G., Conrad C. (2009). Interplay between keratinocytes and immune cells—recent insights into psoriasis pathogenesis. *The International Journal of Biochemistry & Cell Biology*.

[30] Commins S. P., Borish L., Steinke J. W. (2010). Immunologic messenger molecules: cytokines, interferons, and chemokines. *The Journal of Allergy and Clinical Immunology*.

[31] Golden J. B., McCormick T. S., Ward N. L. (2013). IL-17 in psoriasis: implications for therapy and cardiovascular co-morbidities. *Cytokine*.

[32] Buske-Kirschbaum A., Kern S., Ebrecht M., Hellhammer D. H. (2007). Altered distribution of leukocyte subsets and cytokine production in response to acute psychological stress in patients with psoriasis vulgaris. *Brain, Behavior, and Immunity*.

[33] Heidenreich R., Röcken M., Ghoreschi K. (2009). Angiogenesis drives psoriasis pathogenesis. *International Journal of Experimental Pathology*.

[34] Wolk K., Sabat R. (2016). Adipokines in psoriasis: an important link between skin inflammation and metabolic alterations. *Reviews in Endocrine & Metabolic Disorders*.

[35] Griffiths C. E., Barker J. N. (2007). Pathogenesis and clinical features of psoriasis. *Lancet*.

[36] Nickoloff B. J., Xin H., Nestle F. O., Qin J. Z. (2007). The cytokine and chemokine network in psoriasis. *Clinics in Dermatology*.

[37] Li R. C., Krishnamoorthy P., DerOhannessian S. (2014). Psoriasis is associated with decreased plasma adiponectin levels independently of cardiometabolic risk factors. *Clinical and Experimental Dermatology*.

[38] Tilg H., Moschen A. R. (2006). Adipocytokines: mediators linking adipose tissue, inflammation and immunity. *Nature Reviews Immunology*.

[39] Pietrzak A. T., Zalewska A., Chodorowska G. (2008). Cytokines and anticytokines in psoriasis. *Clinica Chimica Acta*.

[40] Strober B., Teller C., Yamauchi P. (2008). Effects of etanercept on C-reactive protein levels in psoriasis and psoriatic arthritis. *The British Journal of Dermatology*.

[41] Furukawa S., Fujita T., Shimabukuro M. (2004). Increased oxidative stress in obesity and its impact on metabolic syndrome. *The Journal of Clinical Investigation*.

[42] Holvoet P., Lee D. H., Steffers M., Gross M., Jacobs D. R. (2008). Association between circulating oxidized low-density lipoprotein and incidence of the metabolic syndrome. *The Journal of the American Medical Association*.

[43] Baran A., Flisiak I., Jaroszewicz J., Swiderska M. (2015). Effect of psoriasis activity on serum adiponectin and leptin levels. *Postepy Dermatologii i Alergologii*.

[44] Vachatova S., Andrys C., Krejsek J. (2016). Metabolic syndrome and selective inflammatory markers in psoriatic patients. *Journal of Immunology Research*.

[45] Ridker P. M. (2016). A test in context: high-sensitivity C-reactive protein. *Journal of the American College of Cardiology*.

[46] Rocha-Pereira P., Santos-Silva A., Rebelo I., Figueiredo A., Quintanilha A., Teixeria F. (2004). The inflammatory response in mild and in severe psoriasis. *The British Journal of Dermatology*.

[47] Kimball A. B., Wu Y. (2009). Cardiovascular disease and classic cardiovascular risk factors in patients with psoriasis. *International Journal of Dermatology*.

[48] Federman D. G., Shelling M., Prodanovich S., Gunderson C. G., Kirsner R. S. (2009). Psoriasis: an opportunity to identify cardiovascular risk. *The British Journal of Dermatology*.

[49] Takahashi H., Tsuji H., Hashimoto Y., Ishida-Yamamoto A., Iizuka H. (2010). Serum cytokines and growth factor levels in Japanese patients with psoriasis. *Clinical and Experimental Dermatology*.

[50] Kaur S., Zilmer K., Leping V., Zilmer M. (2011). The levels of adiponectin and leptin and their relation to other markers of cardiovascular risk in patients with psoriasis. *Journal of the European Academy of Dermatology and Venereology*.

[51] Chodorowska G., Wojnowska D., Juszkiewicz-Borowiec M. (2004). C-reactive protein and α2-macroglobulin plasma activity in medium-severe and severe psoriasis. *Journal of the European Academy of Dermatology and Venereology*.

[52] Coimbra S., Oliveira H., Reis F. (2010). C-reactive protein and leucocyte activation in psoriasis vulgaris according to severity and therapy. *Journal of the European Academy of Dermatology and Venereology*.

[53] Mastroianni A., Minutilli E., Mussi A. (2005). Cytokine profiles during infliximab monotherapy in psoriatic arthritis. *The British Journal of Dermatology*.

[54] Coimbra S., Oliveira H., Reis F. (2009). Circulating levels of adiponectin, oxidized LDL, and C-reactive protein in Portuguese patients with psoriasis vulgaris, according to body mass index, severity and duration of the disease. *Journal of Dermatological Science*.

[55] Coimbra S., Oliveira H., Reis F. (2010). Circulating adipokine levels in Portuguese patients with psoriasis vulgaris according to body mass index, severity and therapy. *Journal of the European Academy of Dermatology and Venereology*.

[56] Deeva J., Mariani S., De Luca C. (2010). Wide-spectrum profile of inflammatory mediators in the plasma and scales of patients with psoriatic disease. *Cytokine*.

[57] Kaur S., Zilmer K., Leping V., Zilmer M. (2012). Comparative study of systemic inflammatory responses in psoriasis vulgaris and mild-to-moderate allergic contact dermatitis. *Dermatology*.

[58] Coimbra S., Oliveira H., Reis F. (2010). Psoriasis therapy and cardiovascular risk factors. *American Journal of Clinical Dermatology*.

[59] Coimbra S., Oliveira H., Reis F. (2010). Interleukin (IL)-22, IL-17, IL-23, IL-8, vascular endothelial growth factor and tumor necrosis factor-*α* levels in patients with psoriasis before, during and after psoralen-ultraviolet A, and narrowband ultraviolet B therapy. *The British Journal of Dermatology*.

[60] Ouchi N., Parker J. L., Lugus J. J., Walsh K. (2011). Adipokines in inflammation and metabolic disease. *Nature Reviews Immunology*.

[61] Blüher M. (2009). Adipose tissue dysfunction in obesity. *Experimental and Clinical Endocrinology & Diabetes*.

[62] Wassink A. M. J., Olijhoek J. K., Visseren F. L. J. (2007). The metabolic syndrome: metabolic changes with vascular consequences. *European Journal of Clinical Investigation*.

[63] Marinou K., Tousoulis D., Antonopoulos A. S., Stefanadi E., Stefanadis C. (2010). Obesity and cardiovascular disease: from pathophysiology to risk stratification. *International Journal of Cardiology*.

[64] Nakanishi S., Yamane K., Kamei N., Nojima H., Okubo M., Kohno N. (2005). A protective effect of adiponectin against oxidative stress in Japanese Americans: the association between adiponectin or leptin and urinary isoprostane. *Metabolism*.

[65] Störk S., Bots M. L., Angerer P. (2007). Low levels of adiponectin predicts worsening of arterial morphology and function. *Atherosclerosis*.

[66] Wolk R., Berger P., Lennon R. J., Brilakis E. S., Davison D. E., Somers V. K. (2007). Association between plasma adiponectin levels and unstable coronary syndrome. *European Heart Journal*.

[67] Kim D. H., Kim C., Ding E. L., Townsend M. K., Lipsitz L. A. (2013). Adiponectin levels and the risk of hypertension: a systematic review and meta-analysis. *Hypertension*.

[68] Fontana V., Faria A. P. d., Oliveira-Paula G. H., Silva P. S., Tanus-Santos J. E., Moreno H. (2014). Effects of angiotensin-converting enzyme inhibition on leptin and adiponectin levels in essential hypertension. *Basic & Clinical Pharmacology & Toxicology*.

[69] Zhang H., Jin X. (2015). Relationship between serum adiponectin and osteoproteregin levels and coronary heart disease severity. *Genetics and Molecular Research*.

[70] Guagnano M. T., Romano M., Falco A. (2003). Leptin increase is associated with markers of the hemostatic system in obese healthy women. *Journal of Thrombosis and Haemostasis*.

[71] Shamsuzzaman A. S., Winnicki M., Wolk R. (2004). Independent association between plasma leptin and C-reactive protein in healthy humans. *Circulation*.

[72] Coimbra S., Catarino C., Santos-Silva A. (2016). The triad psoriasis–obesity–adipokine profile. *Journal of the European Academy of Dermatology and Venereology*.

[73] Gerdes S., Osadtschy S., Rostami-Yazdi M., Buhles N., Weichenthal M., Mrowietz U. (2011). Leptin, adiponectin, visfastin and retinol-binding protein-4 – mediators of comorbidities in the patients with psoriasis?. *Experimental Dermatology*.

[74] Cavusoglu E., Ruwende C., Chopra V. (2006). Adiponectin is an independent predictor of all-cause mortality, cardiac mortality, and myocardial infarction in patients presenting with chest pain. *European Heart Journal*.

[75] Dekker J. M., Funahashi T., Nijpels G. (2008). Prognostic value of adiponectin for cardiovascular disease and mortality. *The Journal of Clinical Endocrinology and Metabolism*.

[76] Aprahamian T. R., Sam F. (2011). Adiponectin in cardiovascular inflammation and obesity. *International Journal of Inflammation*.

[77] Russolillo A., Iervolino S., Peluso R. (2013). Obesity and psoriatic arthritis: from pathogenesis to clinical outcome and management. *Rheumatology*.

[78] Choi C. H. J., Cohen P. (2017). Adipose crosstalk with other cell types in health and disease. *Experimental Cell Research*.

[79] Koh K. K., Park S. M., Quon M. J. (2012). Leptin and cardiovascular diseases: response to therapeutic interventions. *Circulation*.

[80] Scotece M., Conde J., Gómez R. (2012). Role of adipokines in atherosclerosis: interferences with cardiovascular complications in rheumatic diseases. *Mediators of Inflammation*.

[81] Adya R., Tan B. K., Randeva H. S. (2015). Differential effects of leptin and adiponectin in endothelia angiogenesis. *Journal of Diabetes Research*.

[82] Chen Y. J., Wu C. Y., Shen J. L. (2008). Psoriasis independently associated with hyperleptinemia contributing to metabolic syndrome. *Archives of Dermatology*.

[83] Wang Y., Chen J., Zhao Y., Geng L., Song F., Chen H. D. (2008). Psoriasis is associated with increased levels of serum leptin. *The British Journal of Dermatology*.

[84] Woodward L., Akoumianakis I., Antoniades C. (2016). Unravelling the adiponectin paradox: novel roles of adiponectin in the regulation of cardiovascular disease. *British Journal of Pharmacology*.

[85] Ruscica M. A., Baragetti A., Catapano A. L., Norata G. D. (2016). Translating the biology of adipokines in atherosclerosis and cardiovascular diseases: gaps and open questions. *Nutrition, Metabolism, and Cardiovascular Diseases*.

[86] Upadhyay R. K. (2015). Emerging risk biomarkers in cardiovascular diseases and disorders. *Journal of Lipids*.

[87] Mallbris L., Granath F., Hamsten A., Stahle M. (2006). Psoriasis is associated with lipid abnormalities at the onset of skin disease. *Journal of the American Academy of Dermatology*.

[88] Uczniak S., Gerlicz Z. A., Kozłowska M., Kaszuba A. (2016). Presence of selected metabolic syndrome components in patients with psoriasis vulgaris. *Postepy Dermatologii I Alergologii*.

[89] Uyanik B. S., Ari Z., Onur E., Gündüz K., Tanülkü S., Durkan K. (2002). Serum lipids and apolipoprotein in patients with psoriasis. *Clinical Chemistry and Laboratory Medicine*.

[90] Matsuura E., Hughes G. R. V., Khamashta M. A. (2008). Oxidation of LDL and its clinical implication. *Autoimmunity Reviews*.

[91] Kotani K., Koibuchi H., Yamada T., Taniguchi N. (2009). The effects of lifestyle modification on a new oxidized low-density lipoprotein marker, serum amyloid A-LDL, in subjects with primary lipid disorder. *Clinica Chimica Acta*.

[92] Miller Y. I., Shyy J. Y. (2017). Context-dependent role of oxidized lipids and lipoproteins in inflammation. *Trends in Endocrinology and Metabolism*.

[93] Lopez L. R., Buckner T. R., Hurley B. L., Kobayashi K., Matsuura E. (2007). Determination of oxidized low-density lipoproteins (ox-LDL) versus ox-LDL⁄β2GPI complexes for the assessment of autoimmune-mediated atherosclerosis. *Annals of the New York Academy of Sciences*.

[94] Kobayashi K., Kishi M., Atsumi T. (2003). Circulating oxidized LDL forms with beta-2 glycoprotein I: implication as an atherogenic autoantigen. *Journal of Lipid Research*.

[95] Bassi N., Ghirardello A., Iaccarino L. (2007). OxLDL/beta2GPI-anti-oxLDL/beta2GPI complex and atherosclerosis in SLE patients. *Autoimmunity Reviews*.

[96] Tekin N. S., Tekin I. O., Barut F., Sipahi E. Y. (2007). Accumulation of oxidized low-density lipoprotein in psoriatic skin and changes of plasma lipid levels in psoriatic patients. *Mediators of Inflammation*.

